# Air Pollution Exposures and Circulating Biomarkers of Effect in a Susceptible Population: Clues to Potential Causal Component mixtures and mechanisms

**DOI:** 10.1289/ehp.0800194

**Published:** 2009-04-29

**Authors:** Ralph J. Delfino, Norbert Staimer, Thomas Tjoa, Daniel L. Gillen, Andrea Polidori, Mohammad Arhami, Micheal T. Kleinman, Nosratola D. Vaziri, John Longhurst, Constantinos Sioutas

**Affiliations:** 1 Department of Epidemiology, School of Medicine and; 2 Department of Statistics, School of Information and Computer Sciences, University of California, Irvine, Irvine, California, USA; 3 Department of Civil and Environmental Engineering, Viterbi School of Engineering, University of Southern California, Los Angeles, California, USA; 4 Occupational and Environmental Medicine Division; 5 Nephrology and Hypertension Division and; 6 Cardiology Division, Department of Medicine, School of Medicine, University of California, Irvine, California, USA

**Keywords:** cytokines, enzymes, epidemiology, longitudinal data analysis, oxidative stress

## Abstract

**Background:**

Mechanisms involving oxidative stress and inflammation have been proposed to explain associations of ambient air pollution with cardiovascular morbidity and mortality. Experimental evidence suggests that organic components and ultrafine particles (UFP) are important.

**Methods:**

We conducted a panel study of 60 elderly subjects with coronary artery disease living in retirement communities within the Los Angeles, California, air basin. Weekly biomarkers of inflammation included plasma interleukin-6, tumor necrosis factor-α soluble receptor II (sTNF-RII), soluble platelet selectin (sP-selectin), and C-reactive protein (CRP). Biomarkers of erythrocyte antioxidant activity included glutathione peroxidase-1 and superoxide dismutase. Exposures included outdoor home daily particle mass [particulate matter < 0.25, 0.25–2.5, and 2.5–10 μm in aerodynamic diameter (PM_0.25_, PM_0.25–2.5_, PM_2.5–10_)], and hourly elemental and black carbon (EC–BC), estimated primary and secondary organic carbon (OC_pri_, SOC), particle number (PN), carbon monoxide (CO), and nitrogen oxides–nitrogen dioxide (NO_x_–NO_2_). We analyzed the relation of biomarkers to exposures with mixed effects models adjusted for potential confounders.

**Results:**

Primary combustion markers (EC–BC, OC_pri_, CO, NO_x_–NO_2_), but not SOC, were positively associated with inflammatory biomarkers and inversely associated with erythrocyte anti-oxidant enzymes (*n* = 578). PN and PM_0.25_ were more strongly associated with biomarkers than PM_0.25–2.5_. Associations for all exposures were stronger during cooler periods when only OC_pri_, PN, and NO_x_ were higher. We found weaker associations with statin (sTNF-RII, CRP) and clopidogrel use (sP-selectin).

**Conclusions:**

Traffic-related air pollutants are associated with increased systemic inflammation, increased platelet activation, and decreased erythrocyte antioxidant enzyme activity, which may be partly behind air pollutant–related increases in systemic inflammation. Differences in association by particle size, OC fraction, and seasonal period suggest components carried by UFP are important.

Ambient mass concentrations of particulate matter (PM) air pollution < 2.5 μm (PM_2.5_) and < 10 μm (PM_10_) in aerodynamic diameter have been associated with hospital admissions and mortality due to cardiovascular causes in time series studies (Pope and Dockery 2006). Mechanisms involving oxidative stress and inflammation have been proposed to explain these associations (Mills et al. 2007) (Figure 1). In addition, a growing toxicology literature suggests that ultrafine particles (UFP), < 0.1 μm in diameter, may have greater potential to induce oxidative stress and inflammation than larger particles that dominate PM_2.5_ and PM_10_ mass (Ntziachristos et al. 2007). This is likely because compared with larger particles, UFP have a higher airway deposition efficiency, magnitudes higher particle number (PN) concentration and surface area, and higher concentrations of organic components shown to induce oxidative stress responses (Li et al. 2003). The ability of UFP to translocate systemically from pulmonary sites makes them particularly relevant to the cardiovascular effects of inhaled PM (Elder and Oberdörster 2006).

We aimed to improve the characterization of PM exposure in order to yield clues to potentially important pollutant sources and causal component mixtures not otherwise evident with ambient PM_2.5_ and PM_10_ mass, which are regulated by the U.S. Environmental Protection Agency (Delfino et al. 2005) (Figure 1). For example, traffic (a common exposure source of redox active PM) increases spatial variability of UFP (Sioutas et al. 2005). Regional ambient data are thus likely to misrepresent personal exposure. In addition, ambient PM is made up of primary combustion aerosols, photochemically produced secondary organic aerosols, and mechanically generated crustal material. These particle types have different spatial and temporal variability. The organic component mix and size distribution differs as well between these two classes of particulate organic matter, with primary aerosols being more common in UFP and secondary aerosols more common in the accumulation mode (~ 0.1–2.5 μm).

To address these questions, we conducted a panel study with repeated measurements of biomarkers and exposures in 60 elderly individuals with a history of coronary artery disease (CAD), a population potentially susceptible to adverse effects of air pollution (von Klot et al. 2005). We investigated the relationship of intensive measurements of outdoor home air pollutants to changes in circulating bio-markers of inflammation, platelet activation, and antioxidant capacity (Figure 1). These bio-markers may be risk factors for cardiovascular diseases (Espinola-Klein et al. 2007; Pai et al. 2004). We investigated the relative strength of biomarker associations between various PM size fractions. We also investigated differences in associations between exposure markers of traffic-related primary PM compared with secondary PM. Results presented here add a second year of data from 31 subjects to data used in a previous analysis of 29 subjects (Delfino et al. 2008).

## Materials and Methods

### Population

We recruited subjects from four large retirement communities in the Los Angeles (LA), California, air basin. Three were in the San Gabriel Valley, closer to downtown LA and thus closer to traffic sources, and one was further inland in Riverside, California. Eligibility criteria included a confirmed CAD history, ≥ 65 years of age, nonsmoker, and unexposed to environmental tobacco smoke. We clinically evaluated 105 potentially eligible subjects on site. Twenty-one subjects were not eligible, and 20 dropped out or had too few blood draws (< 5 of 12 weeks), leaving 64 subjects. Four subjects had insufficient biomarker data, mostly because of exclusions for frequent infections, leaving 60 subjects ≥ 71 years of age with 5–12 weekly blood draws (*n* = 578) (Table 1). The study protocol was approved by the Institutional Review Board of the University of California, Irvine, and we obtained informed written consent from subjects.

Two communities were studied in 2005–2006 (29 subjects) and two communities were studied in 2006–2007 (31 subjects). We studied subjects in two periods to enhance known contrasts across the LA basin in particle composition and size distribution by season (Sioutas et al. 2005). In each community, we collected 6 weeks of data during a period of higher temperature (July–mid-October), and thus higher photochemical activity and mixing depths, and 6 weeks of data during a cooler period (mid-October–February), with more frequent periods of air stagnation and lower mixing heights (when traffic-related primary air pollutants increase at ground level). Over a 7-month period, each subject was followed weekly in these two 6-week blocks with blood draws for circulating biomarkers of inflammation and antioxidant activity. Subjects completed daily diaries reporting medication use.

### Biomarkers

Venous peripheral blood samples were drawn at the same time of day and day of week (Friday afternoons) and rapidly separated within 30 min into erythrocytes and plasma and frozen at our on-site mobile field laboratory. For the current analysis, we focused on biomarkers that were most informative in the previous analysis of first-year data (Delfino et al. 2008). Plasma samples stored at −80°C were thawed and assayed using 96-well immuno assay kits for the proinflammatory cytokines interleukin-6 (IL-6) and tumor necrosis factor-α (TNF-α) (Quantikine HS, R&D Systems, Minneapolis, MN), soluble TNF-α receptor II (sTNF-RII) (Quantikine, R&D Systems), the acute-phase protein C-reactive protein (CRP) (Zymutest, Hyphen BioMed, Neuville-sur-Oise, France), and a marker of platelet activation, soluble platelet selectin (sP-selectin) (Jurk and Kehrel 2005). Frozen-thawed erythrocyte lysates were assayed spectrophotometrically for activities of two antioxidant enzymes, glutathione peroxidase-1 (GPx-1) and copper–zinc superoxide dismutase (Cu, Zn-SOD) (Cayman Chemical, Ann Arbor, MI), normalized to units per gram of hemoglobin (U/g Hb).

### Exposure assessment

Measurement methods are more thoroughly described in the Supplemental Material (doi:10.1289/ehp.0800194.S1). Hourly outdoor home air pollutants were measured over 9 days before each blood draw as described elsewhere (Arhami et al. 2006; Polidori et al. 2007). These measurements included pollutant gases [carbon monoxide, nitrogen oxides–nitrogen dioxide (NO_x_–NO_2_), ozone], total PN (condensation particle counter model 3785; TSI Inc, Shoreview, MN), PM_2.5_ organic carbon (OC) and elemental carbon (EC) (OC_EC analyzer model 3F; Sunset Laboratory Inc., Tigard, OR), and black carbon (BC) (aethalometer; Magee Scientific, Berkeley, CA). We also measured size-fractionated PM mass with the Sioutas personal cascade impactor sampler (SKC, Inc., Eighty Four, PA) over 24-hr periods from mid-afternoon to mid-afternoon for 5 days before each blood draw (unlike hourly pollutants measured 9 days before). This included particles 0–0.25 μm in diameter (PM_0.25_), accumulation-mode particles 0.25–2.5 μm in diameter (PM_0.25–2.5_), and coarse mode particles 2.5–10 μm in diameter (PM_2.5–10_). We refer to PM_0.25_ as “quasi-ultrafine” because the upper size cutpoint for the ultrafine mode has varied from 0.1 to 0.2 μm, depending on locations and seasons. UFP are traditionally defined as those originating mostly from fresh emission sources and accounting for > 90% of the number-based particle concentrations (Sioutas et al. 2005). A major fraction of accumulation-mode PM originates from the ultrafine mode. This is unlike coarse particles and fine particles (PM_2.5_, or accumulation plus ultrafine), which are naturally divided by a cutpoint of 2.5 μm and have clearly different origins.

We estimated outdoor secondary OC (SOC) and primary OC (OC_pri_) from total OC as detailed in our recent publication (Polidori et al. 2007) and summarized in the Supplemental Material (doi:10.1289/ehp.0800194.S1). OC_pri_ is representative of particles emitted directly from combustion sources (mostly fossil fuels in the LA basin), whereas SOC represents semivolatile and low-volatile products of photochemical reactions involving reactive organic gases from anthropogenic and biogenic sources. The study average outdoor SOC accounted for 34% and 44% of total OC in the cooler and warmer phases, respectively.

### Analysis

We used linear mixed-effects models to analyze relationships of biomarkers to air pollutant exposures (Verbeke and Molenberghs 2001). Because within-individual repeated measures of outcomes are correlated, random effects were estimated at the subject level, nested within phase and community. The covariance structure observed from empiric variograms was representative of an autoregressive-1 correlation, and models were fit as such.

Using mean centered exposures, we adjusted for between-subject group and between-phase exposure effects. Thus, the interpretation of reported estimates is at the subject level [see Supplemental Material (doi:10.1289/ehp.0800194.S1)].

To assess more acute versus cumulative exposure–response relationships, we evaluated last 24-hr averages of air pollutants (1 day) as well as cumulative exposures up to 9 days (or 5 days for particle mass) before the blood draw. We chose a set of averaging times that skipped over averages by 1 day to simplify the presentation while still presenting a view of associations across the span of averaging times (1-day, 3-day, 5-day, 7-day, and 9-day averages).

We decided *a priori* to exclude person-weeks with acute infectious illnesses, given their known impact on measured biomarkers. We controlled for temperature at the same averaging time as the air pollutant.

We hypothesized *a priori* that medication variables known to influence inflammation and oxidative stress would act as effect modifiers. This included 3-hydroxy-3-methylglutaryl coenzyme A (HMG CoA) reductase inhibitors (statins), and angiotensin-converting enzyme (ACE) inhibitors or angiotensin II receptor blockers (ARB). We also tested effect modification of sP-selectin associations by clopidogrel, a platelet aggregation inhibitor.

We tested differences in association by seasonal phase of study to gain clues regarding underlying differences in potentially important air pollutant components. In addition, given the known differences in air pollution in the San Gabriel Valley (three communities) compared with Riverside (one community), we tested differences in association between these regions. We planned these analyses in advance by designing the study to follow subjects during two seasonal phases and in different regions, known factors leading to differences in pollutant components and particle size distribution. All interactions (medications, phase, and group) were tested in product term models and all stratified results come from these models including all data.

Associations were more strongly positive in the San Gabriel Valley (44 subjects) than in Riverside (16 subjects) for sTNF-RII and sP-selectin [see Supplemental Material (doi:10.1289/ehp.0800194.S1)]. To simplify model presentation, we show results for sTNF-RII and sP-selectin for subjects in the three San Gabriel Valley communities.

We examined residual diagnostics to investigate deviations from standard linear mixed-model assumptions (functional form of independent variables and covariance assumptions) and the presence of influential observations. Four influential high outliers for IL-6 > 10 pg/mL were reset to 10 pg/mL, and one extreme influential outlier for sP-selectin (221 ng/mL) was removed to obtain more representative estimates of association. Residuals for both CRP and TNF-α exhibited a highly skewed distribution, primarily due to a cluster of subjects in the upper quartile of biomarker concentrations, and 2–3 high outliers > 3 SD above the mean. Outliers were reset to the next highest values, and secondary subgroup analyses were conducted among subjects in the upper quartile of mean CRP versus the lower three quartiles. Although this analysis was clearly data driven, similar subgroup analyses have been previously reported (Dubowsky et al. 2006; Rückerl et al. 2006). Mixed-models analyses for both CRP and TNF-α were stratified as such to show differential risk by chronic inflammation and to express results for both variables in their measured units.

To identify influential subject clusters, we tested random slopes models as well as individual autoregressive models. Through this exploratory data analysis, we found that five subjects in erythrocyte Cu, Zn-SOD models and three subjects in erythrocyte GPx-1 models formed highly influential clusters with positive associations between air pollutants and biomarkers. One subject was a positive responder for both biomarkers [see Supplemental Material (doi:10.1289/ehp.0800194.S1)]. Below, we present results for secondary analyses excluding these influential subjects. Because these results stem from a sensitivity analysis, the reported results should be interpreted conservatively.

## Results

### Descriptive statistics

Descriptive data for bio-marker measurements are shown in Table 2. Table 3 gives descriptive statistics for exposures by phase of study. Exposures were generally similar across the two phases, except for notably higher concentrations of OC_pri_, PN, and NO_x_ in phase 2 (colder phase), and higher concentrations of SOC and O_3_ in phase 1 (warmer phase). High outdoor PM_0.25_ relative to PM_2.5_ are likely attributable to large impacts of local traffic in the LA basin compared with the eastern half of the nation with much larger contributions to PM_2.5_ from accumulation-mode sulfate aerosols. Table 4 shows exposure correlations for combined phases. EC, BC, OC_pri_, NO_x_, and CO were strongly correlated with each other, likely because they are products of fossil fuel combustion. These correlations were stronger in phase 2 than in phase 1 (data not shown). PN and PM_0.25_ concentrations were moderately correlated with these combustion-related pollutants, and these correlations were stronger in the three San Gabriel Valley communities closer to traffic sources than in Riverside (data not shown). There is a stronger correlation between PM_0.25_ and PM_2.5–10_ (coarse particles) than between PM_0.25_ and PM_0.25–2.5_ because PM_0.25_ and coarse particles come from primary traffic sources in our study region. Whereas PM_0.25_ is primarily a product of fresh emissions, PM_0.25–2.5_ is a product of aging and photo-chemical reactions.

### Regression analysis

Many positive associations were found for IL-6, sP-selectin, sTNF-RII, TNF-α, and CRP with markers of traffic-related air pollution (EC, OC_pri_, BC, NO_x_, and CO). We also found inverse associations of Cu, Zn-SOD and GPx-1 with the same pollutants. However, this was found only in the restricted subset of 55 (Cu, Zn-SOD) and 57 subjects (GPx-1) as presented below, whereas models including all 60 subjects were mostly nonsignificant [see Supplemental Material (doi:10.1289/ehp.0800194.S1)]. To simplify the presentation, we focus here on two biomarkers of inflammation (IL-6 and sTNF-RII) and present results for TNF-α and CRP online [see Supplemental Material (doi:10.1289/ehp.0800194.S1)]. We also present results for two key markers of primary combustion (EC and OC_pri_) that were strongly correlated with the other markers not shown (BC, NO_x_, and CO). We present pollutant averaging times here that best represent associations across the span of time rather than the full set of selected averaging times. In many but not all cases, associations were strongest for longer-term averages out to the last 5 days and, in some cases, 9 days. Regression results for all pollutants and all selected lag averages are shown online [see Supplemental Material (doi:10.1289/ehp.0800194.S1)].

Biomarkers of systemic inflammation (IL-6 and sTNF-RII), but not sP-selectin, were more strongly and significantly associated with quasi-ultrafine PM_0.25_ than larger-size fractions (Figure 2). We also found inverse associations of Cu, Zn-SOD and GPx-1 with PM_0.25_ that were somewhat stronger than larger-size fractions.

Across all biomarkers, we also found consistently stronger associations for OC_pri_ than for SOC, and this was generally reflected by confidence intervals (CIs) for total OC that usually crossed zero (Figure 3).

Associations were generally stronger in phase 2 than in phase 1 for IL-6, Cu, Zn-SOD, and sP-selectin (Figure 4). The panels were marginally different across phases, because subjects were followed in two phases with few exceptions (5 of 60 subjects). In models restricted to 55 subjects with data in both phases, phase differences were nearly unchanged (data not shown).

Furthermore, associations were stronger among subjects not taking statins for sTNF-RII and stronger among subjects not taking clopidogrel for sP-selectin (Figure 5). There were few consistent pollutant interactions with ACE/ARB.

As previously reported and discussed for year 1 data (Delfino et al. 2008) but not presented here, regression coefficients for ozone had opposite signs compared with other pollutants [see Supplemental Material (doi:10.1289/ehp.0800194.S1)] and were completely confounded by markers of primary combustion (EC, BC, OC_pri_, CO, NO_x_) with which O_3_ was inversely correlated (Table 4).

## Discussion

Our results are largely consistent with recent repeated-measures studies showing associations between ambient air pollution and bio-markers of systemic inflammation in healthy young adults (Chuang et al. 2007) and susceptible subjects with CAD (Dubowsky et al. 2006; Rückerl et al. 2006, 2007a; 2007b; Yue et al. 2007). We extended these previous findings with data from intensive home exposure assessments and modeling that provided clues to potentially causal pollutant components. This is also the first study to show adverse effects of air pollutants on erythrocyte anti-oxidant enzymes.

We focused on elucidating the role of pollutants closely associated with traffic, including EC and PM_0.25_. This was accomplished with extensive measurements of exposures in the immediate outdoor community microenvironments of subjects, including size-fractionated PM, OC fractions, and measurements across seasons that helped us characterize differences in response potentially due to particle size distribution and chemical composition. The approach likely enabled us to detect stronger associations with PM_0.25_ than PM_0.25–2.5_ (Figure 2). Such differences in these two particle-size fractions of regulated fine PM (PM_2.5_) have not been as clearly demonstrated in previous panel studies that have relied on central site data. This finding may be attributable to the higher deposition fraction of the unmeasured UFP fraction (PM_0.1_) of PM_0.25_ than accumulation-mode particles and the ability of UFP to translocate systemically to potentially induce oxidative stress and inflammation (Elder and Oberdörster 2006; Li et al. 2003).

The approach also enabled us to demonstrate for the first time that associations of IL-6, sP-selectin, and SOD with PM markers of primary combustion (EC, OC_pri_), PN, and PM_0.25_ were stronger in a cooler 6-week period (phase 2) than a warmer 6-week period (phase 1) (Figure 4). We did not find any positive associations with SOC (Figure 3), with most regression coefficients being negative and nonsignificant at *p* < 0.05. OC_pri_ and PN concentrations were higher in cooler months (typically characterized by air stagnation and lower secondary particle formation). Interestingly, concentrations of other pollutants also more strongly associated with biomarkers in the cooler phase (EC, BC, and PM_0.25_) were not higher, suggesting that differences in particle composition or size distribution were important, perhaps as better reflected by OC_pri_ and PN, respectively. This is an important finding because particle mass alone does not provide sufficient information about composition or sources. It is conceivable, for example, that our findings for PM_0.25_ and PN are attributable partly to nanoparticles. It has been shown that particles 6–12 nm were much higher in the winter than in the summer near a Los Angeles freeway, but larger particles 50–100 nm showed the opposite trend (Zhu et al. 2004). The potential importance of traffic-related particles is supported by stronger positive associations for sTNF-RII and sP-selectin in the San Gabriel Valley communities closer to traffic sources than in the Riverside community. Associations were also generally stronger for OC_pri_ (Figure 3) than for PM_0.25_ (Figure 2). OC_pri_ and related EC are mostly associated with UFP (Li et al. 2003). Because PM_0.25_ includes some accumulation-mode particles, it likely represents both fresh and aged traffic-related particles. Based on these results, both primary organic components and quasi-ultrafine or smaller-size fractions appear to be important.

Semivolatile organic components associated with particles may also have been important, given that biomarker associations were similarly robust for the correlated gases CO and NO_x_ [see Supplemental Material (doi:10.1289/ehp.0800194.S1)]. This included generally stronger associations with gases in phase 2 than in phase 1 when NO_x_ concentrations were lower. These gases were unlikely causal at the observed low concentrations (Devlin et al. 1999; Thom et al. 2006) but instead served as markers for other traffic emission components.

Other findings support the hypothesis that effects of air pollution on cardiovascular health are secondary to proinflammatory properties of redox active and other pollutant components (Delfino et al. 2005). Associations for sTNF-RII were stronger in subjects not taking statins, which have anti-inflammatory properties. These findings are consistent with reports of weaker associations between air pollutants and CRP among statin users in two other panel studies of susceptible elderly subjects (Dubowsky et al. 2006; Rückerl et al. 2006).

**Figure 1:**
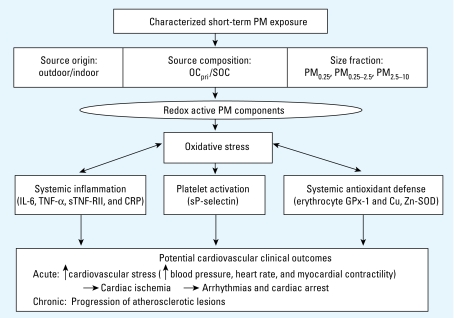
Pathways from PM exposure to measured changes in systemic biomarkers and to hypothesized adverse cardiovascular health effects. The different source characteristics and particle-size fractions we measured represent different types and concentrations of redox active components, including organic chemicals (we measured OC), that may lead to the production of reactive oxygen species in various cells of the lungs, blood, and vascular tissues. This will induce oxidative stress and then antioxidant responses from enzymes involved in oxidant defense, either by interacting with a relatively fixed pool of erythrocyte enzymes (we measured GPx-1 and Cu, Zn-SOD) or through induction of genes coding these enzymes in nucleated cells. Proinflammatory effects (we measured IL-6, TNF-α, sTNF-RII, and CRP) then occur at higher levels of exposure-induced oxidative stress when antioxidant defenses are overwhelmed (Li et al. 2003). This is expected to lead to upregulation of adhesion molecules on vascular endothelium, circulating leukocytes, and platelets (we measured sP-selectin). Induction of endothelial dysfunction, inflammation, and thrombosis by these mechanisms would increase the risk of acute adverse cardiovascular outcomes. Repeated acute insults such as these likely contribute to atherogenesis.

**Figure 2:**
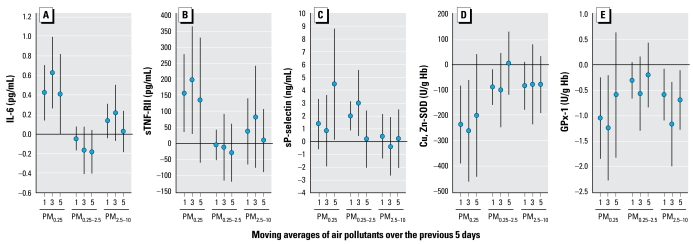
Associations of biomarkers with outdoor size-fractionated particle mass on days 1, 3, and 5. Expected change in the biomarker (adjusted coefficient and 95% confidence interval) corresponds to an interquartile range change in the air pollutant (Table 3). Models for sTNF-RII and sP-selectin are restricted to 44 subjects living in the San Gabriel Valley.

**Figure 3:**
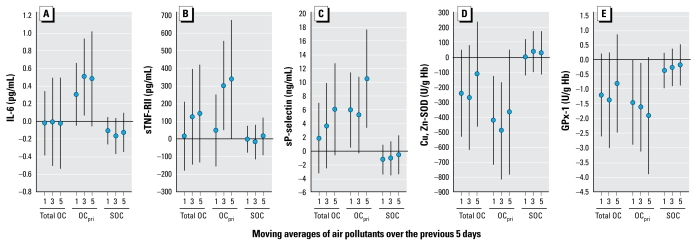
Associations of biomarkers with indoor and outdoor OC on days 1, 3, and 5: differences by OC_pri_ and SOC fractions. Expected change in the biomarker (adjusted coefficient and 95% CI) corresponds to an interquartile range change in the air pollutant (Table 3). Models for sTNF-RII and sP-selectin are restricted to 44 subjects living in the San Gabriel Valley.

**Figure 4:**
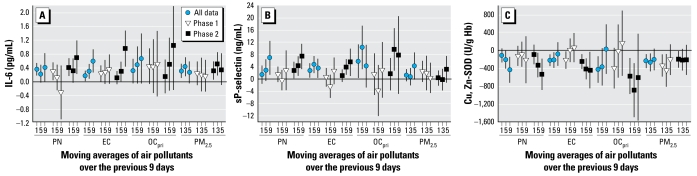
Associations of biomarkers with outdoor air pollutants on days 1, 5, and 9: differences by phase of study. (*A*) IL-6 (pg/mL). (*B*) sP-selectin (ng/mL). (*C*) Cu, Zn-SOD (U/g Hb). Expected change in the biomarker (adjusted coefficient and 95% CI) corresponds to an interquartile range change in the air pollutant (Table 3). Phase 1 is a warmer period of greater photochemical activity, and phase 2 is a cooler period of greater air stagnation. Results for sP-selectin are for 44 subjects living San Gabriel Valley.

**Figure 5:**
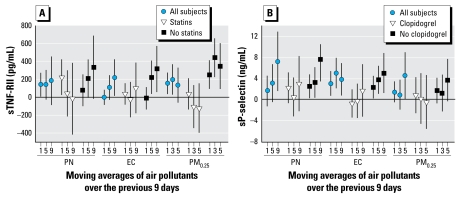
Associations of biomarkers with outdoor air pollutants on days 1, 3, 5, and 9: differences by medication use among 44 subjects living in the San Gabriel Valley. (*A*) sTNF-RII (pg/mL). (*B*) sP-selectin (ng/mL). Expected change in the biomarker (adjusted coefficient and 95% CI) corresponds to an interquartile range change in the air pollutant (Table 3).

Our new finding for sP-selectin is consistent with a panel study showing an association of ambient UFP with another platelet activation marker (soluble CD40 ligand) in people with CAD (Rückerl et al. 2007b). Our study is the first to show a protective effect of clopidogrel. This finding supports the plausibility of a pollutant effect on platelet activation, because this medication blocks platelet aggregation and is associated with decreased sP-selectin (Xiao and Théroux 2004). Our findings are relevant to the potential for air pollution to affect CAD, because sP-selectin activates both leukocytes and endothelial cells and induces adhesion of leukocytes to platelets and to endothelial cells (Jurk and Kehrel 2005). Therefore, if air pollutants acutely activate platelets as suggested by our finding, this could increase the risk of a potentially fatal thrombotic event in the coronary arteries (Figure 1). Platelet selectin is also critical to the development of neointimal formation after arterial injury (Wang et al. 2005). Potentially relevant findings by two epidemiologic studies are evidence of increased risks of athero sclerosis development with exposure to traffic-related air pollution near the home (Hoffmann et al. 2007; Künzli et al. 2005).

Other novel findings are the inverse associations between air pollutants and two anti-oxidant enzymes (GPx-1 and Cu, Zn-SOD). Experimental results show that urban UFP can induce a positive antioxidant response represented by hemoxygenase-1 in epithelial and macrophage cell cultures (Li et al. 2003). However, erythrocytes do not have nuclei and thus have a relatively fixed amount of antioxidant enzymes after maturation from reticulocytes. The findings for GPx-1 and Cu, Zn-SOD in most of the elderly subjects studied suggest enzyme inactivation within erythrocytes by pollutant components or PM_0.25_. There is experimental evidence to support this hypothesis (Hatzis et al. 2006; Pigeolet et al. 1990; Shinyashiki et al. 2008) as well as evidence showing that quasi-UFP ≤ 0.2 μm in diameter and nanoparticles, but not larger particles, freely enter the erythrocyte (Rothen-Rutishauser et al. 2006). This may be a clue to an important pathway involving primarily UFP (Elder and Oberdörster 2006) and related organic and inorganic components that may enter the circulation to then target erythrocytes as well as other cells.

Erythrocytes are critical in protecting the body against oxidative stress (Tsantes et al. 2006). Therefore, it is conceivable that erythrocyte antioxidant enzyme inactivation is partly responsible for pollutant-related increase in biomarkers of inflammation and thrombosis. This is supported by our finding of within-subject inverse associations of IL-6 with GPx-1, and sP-selectin with Cu, Zn-SOD in mixed models. For an interquartile range decrease in GPx-1 of 10.4 U/g hemoglobin, IL-6 increased 0.25 pg/mL (95% CI, −0.03 to 0.53) or 10% of mean IL-6. Similarly, for an interquartile range decrease in SOD of 2,026 U/g hemoglobin, sP-selectin increased 5.8 ng/mL (95% CI, 3.3 to 8.3), or 13% of mean sP-selectin. Biomarkers of inflammation were generally positively associated with each other, and GPx-1 was positively associated with Cu, Zn-SOD (data not shown). Furthermore, erythrocyte antioxidant enzyme inactivation may modulate the putative effects of air pollutants on endothelial dysfunction. Erythrocytes have been shown to protect cultured endothelial cells against oxidant damage. Inhibitors of either the erythrocyte glutathione system or membrane transport of superoxide into erythrocytes significantly reduced this protection (Richards et al. 1998).

A small subset of subjects showed positive GPx-1 and Cu, Zn-SOD associations with air pollutants. Compared with the 53 negative responders, these seven subjects showed no notable differences in the distributions of characteristics listed in Table 1 except that none took clopidogrel and only one had a history of myocardial infarction. We speculate that these might be healthier subjects because of their ability to increase antioxidant enzyme activity, perhaps by mounting a rapid bone marrow response to PM exposure, as suggested in several experimental studies (Goto et al. 2004; Mukae et al. 2001), including increased reticulocytes (Rivero et al. 2005). In mature erythrocytes, activities of Cu, Zn-SOD and GPx-1 are steadily eroded by oxidative and nitrosative stress as they age. Consequently individuals with robust erythropoietic activity and greater proportion of newly released erythrocytes would be expected to have higher erythrocyte Cu, Zn-SOD and GPx-1 activities than sicker individuals.

Study limitations include the following: Despite the biological plausibility, effect modification by medication use could have been secondary to other unmeasured characteristics of subjects. The home exposure data may be subject to exposure error because of differences with personal exposure. However, subjects stayed at home 88% of the time (from diary data). Although we believe that the present exposure data represent key sources and components, we cannot link exposure to specific sources, nor can we identify specific component classes such as polycyclic aromatic hydrocarbons as being responsible for associations. Nevertheless, the major source of fossil fuel emissions in the LA basin is motor vehicle exhaust, and because EC, BC, OC_pri_, CO, and NO_x_ are linked to these emissions, our data suggest that vehicular pollutants are behind the reported associations.

## Conclusion

Our results suggest that pollutant components linked to emission sources of primary PM_2.5_ OC, quasi-UFP (PM_0.25_), and PN concentrations are associated with increased systemic inflammation, platelet activation, and decreased circulating erythrocyte antioxidant enzyme activity in elderly people with CAD. Inactivation of antioxidant enzymes may be one mechanism of air pollutant–related increases in systemic inflammation. These effects may be partly behind reported morbidity and mortality associations with ambient PM_2.5_ mass concentrations (Pope and Dockery 2006). Stronger associations during the cooler phase of study, despite similar PM_0.25_ mass concentrations in cooler and warmer phases, further support the view that the greatest impacts on systemic responses may be attributable to nanoparticles not adequately represented by the present particle mass measurements as well as to unmeasured toxic air pollutants that increase near ground level in the winter. Our related experimental work using particles collected in the LA air basin at the Southern California Particle Center suggests that this might include redox active and electrophilic organic components of traffic exhaust particles in the ultrafine range (Araujo et al. 2008; Gong et al. 2007; Li et al. 2003; Ntziachristos et al. 2007; Shinyashiki et al. 2008).

## Figures and Tables

**Table 1:** Characteristics of subjects (*n* = 60).

Characteristic	Value
Age (years)	84.1 ± 5.60
Body mass index (kg/m^2^)	26.8 ± 3.87
Sex	
Male	34 (56.7)
Female	26 (43.3)
Cardiovascular history	
Confirmation of CAD^a^	
Myocardial infarction	27 (45.0)
Coronary artery bypass graft or angioplasty	20 (33.3)
Positive angiogram or stress test	10 (16.7)
Clinical diagnosis^b^	3 (5.0)
Current angina pectoris	18 (30.0)
Congestive heart failure	13 (21.7)
Hypertension	42 (70.0)
Hypercholesterolemia (by history)	43 (71.7)
Medications	
ACE inhibitors and angiotensin II receptor antagonists	24 (40.0)
HMG-CoA reductase inhibitors (statins)	31 (51.7)
Clopidogrel bisulfate (Plavix)^c^	21 (35.0)

Values are mean ± SD or no. (%).

a Each category is hierarchical and excludes being in the above diagnostic category.

b Includes subjects with anginal symptoms relived with nitrates plus echocardiogram and ECG evidence of past infarct.

c Eight were also taking coumadin.

**Table 2:** Biomarker concentrations (578 measurements).

Biomarker^a^	Mean ± SD	Median (min/max)
IL-6 (pg/mL)	2.42 ± 1.85	1.92 (0.25/10.0)
TNF-α (pg/mL)^b^	1.92 ± 1.64	1.41 (0.5/14.3)
sTNF-RII (pg/mL)	3,610 ± 1,489	3,235 (657/11,584)
sP-selectin (ng/mL)	45.0 ± 16.6	42.5 (6.0/119.9)
CRP (ng/mL)^c^	2,434 ± 3,181	1,403 (250/26,799)
Cu, Zn-SOD (U/g Hb)	4,459 ± 1,688	4,288 (669/13,138)
GPx-1 (U/g Hb)	19.2 ± 7.6	17.8 (5.5/50.7)

Abbreviations: max, maximum; min, minimum.

a Excludes observations for weeks when there was a reported infection.

b Values of TNF-α < 0.5 could not be quantified and were set to 0.5.

c Values of CRP < 250 could not be quantified and were set to 250.

**Table 3:** Descriptive statistics of outdoor air pollutant measurements.

	Phase 1				Phase 2				
Exposure (24-hr average)	No. (missing)	Mean ± SD	IQR	Min/max	No. (missing)	Mean ± SD	IQR	Min/max	IQR overall^a^
Outdoor hourly PM									
EC (μg/m^3^)	161 (19)	1.45 ± 0.52	0.706	0.53/3.01	139 (34)	1.55 ± 0.71	1.08	0.24/3.94	0.87
OC (μg/m^3^)	164 (16)	7.90 ± 4.65	4.43	2.32/27.26	141 (32)	9.25 ± 4.33	7.62	2.51/17.72	7.59
BC (μg/m^3^)	180 (0)	1.59 ± 0.63	0.86	0.38/3.37	172 (1)	1.76 ± 0.91	1.24	0.30/5.11	1.03
OC_pri_ (μg/m^3^)	161 (19)	4.36 ± 2.14	2.91	1.28/10.04	139 (34)	6.03 ± 3.53	6.25	0.99/13.64	4.04
SOC (μg/m^3^)	161 (19)	3.48 ± 3.40	2.03	0.28/18.74	139 (34)	3.12 ± 1.62	2.50	0.00/6.91	2.42
PN/cm^3^	133 (47)	10,243 ± 4,438	6,526	1,441/24,302	152 (21)	14,851 ± 6,490	8,631	3,297/31,264	7,354

Outdoor PM mass									
PM_0.25_ (μg/m^3^)	111 (9)	10.27 ± 3.69	4.79	3.16/22.82	106 (7)	9.25 ± 4.48	5.60	2.46/30.05	7.00
PM_0.25–2.5_ (μg/m^3^)	115 (5)	12.23 ± 6.39	9.54	1.64/27.78	111 (2)	10.47 ± 11.70	9.95	0.98/66.77	10.58
PM_2.5–10_ (μg/m^3^)	110 (10)	11.45 ± 4.65	5.32	1.15/23.41	107 (6)	7.25 ± 4.39	5.22	0.30/24.63	5.46

Outdoor hourly gases									
NO_2_ (ppb)	179 (1)	26.41 ± 11.97	19.17	4.52/59.83	172 (1)	28.34 ± 11.80	17.57	3.78/55.74	14.30
NO_x_ (ppb)	179 (1)	37.17 ± 22.44	28.13	3.70/112.43	172 (1)	53.86 ± 36.14	50.9	4.26/188.0	41.60
CO (ppm)	173 (7)	0.50 ± 0.25	0.36	0.11/1.30	162 (11)	0.58 ± 0.35	0.52	0.01/1.68	0.51
O_3_ (ppb)	179 (1)	33.30 ± 11.40	15.52	8.04/76.35	170 (3)	20.62 ± 8.04	10.8	6.17/44.90	16.09

Abbreviations: IQR, interquartile range; max, maximum; min, minimum.

a This overall IQR was used to estimate the expected change in the biomarker (coefficient and 95% CI) from exposure to the air pollutant.

**Table 4:** Outdoor exposure correlation matrix.^a^

	OC	BC	OC_pri_	SOC	PN	PM_0.25_	PM_0.25–2.5_	PM_2.5–10_	NO_2_	NO_x_	CO	O_3_
EC	0.61	0.89	0.97	−0.03	0.50	0.54	0.31	0.36	0.80	0.82	0.78	−0.39
OC	1.00	0.63	0.65	0.72	0.27	0.41	0.33	0.33	0.55	0.46	0.59	−0.05
BC		1.00	0.88	0.07	0.40	0.52	0.43	0.44	0.88	0.83	0.79	−0.38
OC_pri_			1.00	0.01	0.47	0.55	0.33	0.36	0.78	0.79	0.75	−0.36
SOC				1.00	−0.08	0.09	0.16	0.15	0.07	−0.09	0.11	0.26
PN					1.00	0.36	−0.12	0.06	0.48	0.63	0.45	−0.38
PM_0.25_						1.00	0.17	0.35	0.56	0.51	0.54	0.01
PM_0.25–2.5_							1.00	0.60	0.19	0.01	0.13	0.08
PM_2.5–10_								1.00	0.32	0.18	0.26	0.06
NO_2_									1.00	0.88	0.79	−0.42
NO_x_										1.00	0.82	−0.53
CO											1.00	−0.29

a All exposures are mean-centered by group and phase [see Supplemental Material (doi:10.1289/ehp.0800194.S1)].
