# Correction: Air Pollution Exposures and Circulating Biomarkers of Effect in a Susceptible Population: Clues to Potential Causal Component Mixtures and Mechanisms

**DOI:** 10.1289/ehp.0800194-erratum

**Published:** 2010-01-01

The authors report an error in Figure 2 of “Air Pollution Exposures and Circulating Biomarkers of Effect in a Susceptible Population: Clues to Potential Causal Component Mixtures and Mechanisms” [Environ Health Perspect 117:1232–1238 (2009)]. In “Materials and Methods,” they noted that “four influential high outliers for IL-6 > 10 pg/mL were reset to 10 pg/mL”; however, the analysis shown in Figure 2A for inter­leukin (IL)-6 included these outliers without them having been reset to 10 pg/mL. In all other figures showing results for IL-6 and in Supplemental Material (doi:10.1289/ehp.0800194.S1 via http://dx.doi.org/), the outliers were correctly reset to 10 pg/mL. The IL-6 outliers are themselves valid measurements, and the results in both cases do not change the finding that the association of IL-6 with PM0.25 (particulate matter < 0.25 µm in aerodynamic diameter) is stronger than associations with larger particle size fractions.

